# Immunodeficiency due to a novel variant in *PIK3CD*: a case report

**DOI:** 10.1186/s12969-023-00859-y

**Published:** 2023-07-20

**Authors:** Niloofar Shashaani, Zahra Chavoshzadeh, Leila Ghasemi, Shabnam Hajiani Ghotbabadi, Sara Shiari, Samin Sharafian, Reza Shiari

**Affiliations:** 1 Department of Pediatric Rheumatology, Shahid Beheshti University of Medical Sciences, Tehran, Iran; 2 Department of Allergy and Clinical Immunology, Mofid Children’s Hospital, Shahid Beheshti University of Medical Sciences, Tehran, Iran; 3 Rheumatology Department, Shiraz University of Medical Sciences, Shiraz, Iran; 4 Division of Oncology, Masih Daneshvari Hospital, Shahid Beheshti University of Medical Sciences, Tehran, Iran

**Keywords:** Combined Immunodeficiency syndrome, Activated phosphoinositide 3-kinase delta syndrome (APDS), Phosphoinositide 3-kinase *(PI3K)*

## Abstract

**Background:**

Primary immunodeficiencies are immunological disorders caused by gene mutations involved in immune system development and activation. Recently, activated phosphoinositide 3-kinase delta syndrome (APDS) due to mutations in the phosphoinositide 3-kinase (*PI3K)*, *phosphatidylinositol-4, 5-bisphosphate 3-kinase, catalytic subunit delta gene (PIK3CD*), and phosphoinositide 3-kinase regulatory subunit 1 *(PIK3R1*) genes have been reported to induce a combined immunodeficiency syndrome leading to senescent T cells, lymphadenopathy, and immunodeficiency. The exact diagnosis of these deficiencies is essential for treatment and prognosis. In recent years, targeted treatment with selective PI3Kd inhibitors has had a significant effect on controlling the symptoms of these patients.

**Case presentation:**

In this case report, we represent a 27-month-old girl with recurrent fever, an increased level of inflammatory markers, and erythema nodosum, who was referred to the rheumatology clinic. In the course of evaluations, because of the lack of clinical improvement with usual treatments, and a history of frequent respiratory infections, combined immunodeficiency was diagnosed in the immunological investigations. Moreover, whole-exome sequencing was performed for her.

**Conclusion:**

The genetic analysis found a novel variant of *PIK3CD* (c.1429 G > A) in the patient. Following daily antibiotic prophylaxis and monthly IV therapy, the patient’s frequent infections and fevers were controlled.

## Introduction

Primary immune deficiency (PID) includes a wide variety of diseases in which the immune system is impaired or incapable of performing its functions normally, putting the individual at risk for infections, cancer, and autoimmune conditions. It has been recognized genetically that there are more than 480 distinct disorders, and new diseases are continually being discovered [1]. In most cases, primary immunodeficiency disorders are inherited from parents, but some are acquired [2, 3].There are different reports on the prevalence of PID worldwide. It is estimated that about 1 per 10,000 people are involved with PID [4, 5].

The p110δ catalytic subunit widely produced in immune cells is encoded by the phosphatidylinositol-4, 5-bisphosphate 3-kinase, catalytic subunit delta gene (*PIK3CD*). The mutation of this gene can result in lymphadenopathy and combined immunodeficiency. Mutations that cause this gene gain or loss of function have been reported to induce immunodeficiency in different ways [6].

PIDs can occur at any age, and diagnosing them accurately and promptly necessitates high suspicion and specific testing. A wide variety of clinical manifestations occur with PIDs, but most increase the risk of infection. Many PIDs result in frequent infections and may go unnoticed in general care. Diagnosing and treating diseases as early as possible is imperative to avoid considerable disease-related morbidity and improve patient outcomes [7, 8]. In this case report, we presented a girl with a novel mutation in the PIK3CD gene that has not been reported until now, to the best of our knowledge.

## Case presentation

A 27-month-old girl was referred due to recurrent fever, arthritis, and erythema nodosum to the rheumatology clinic of Mofid Children’s Hospital (Tehran, Iran). She was the second alive child of consanguineous marriage (G2P2L2) with a 3800-gram birth weight and a history of neurodevelopmental delays in walking (at the age of 21 months) and speaking (at the age of 24 months). There is no positive point in the family history, except for the mother, who had hypothyroidism and a history of frequent respiratory infections in childhood.

She had a history of several hospital admissions after her birth. First, she was admitted at two months old with a fever and high levels of ESR and CRP. She received antibiotics for five days and was discharged with no signs or symptoms but still had high ESR and CRP.

At 8 months old, she was evaluated due to failure to thrive (FTT). During patient examinations, the low heart rate, muffled heart sound, and massive pericardial effusion were found in her echocardiography. The pericardial effusion caused her to be hospitalized for 16 days in the intensive care unit (ICU). Pericardial effusion was tapped without any infectious resources. Pericardial effusion was treated with prednisolone, and she was discharged in good condition. Two months later, she was hospitalized again with recurrent pericardial effusion for 19 days. In evaluations, she had hypothyroidism and a little pericardial effusion. She was treated with dexamethasone, ibuprofen, and levothyroxine.

Fourth and fifth hospitalizations occurred due to high-grade fever and elevated ESR and CRP levels at the 13th and 18th months of old, respectively.

She got COVID-19 at 24 months old and was admitted to the hospital for the 6th time because of her past medical history. She had only a fever as a COVID-19 manifestation. After 4 months, she got COVID-19 again and had gastrointestinal manifestations without any adverse effects.

**Fig. 1:**
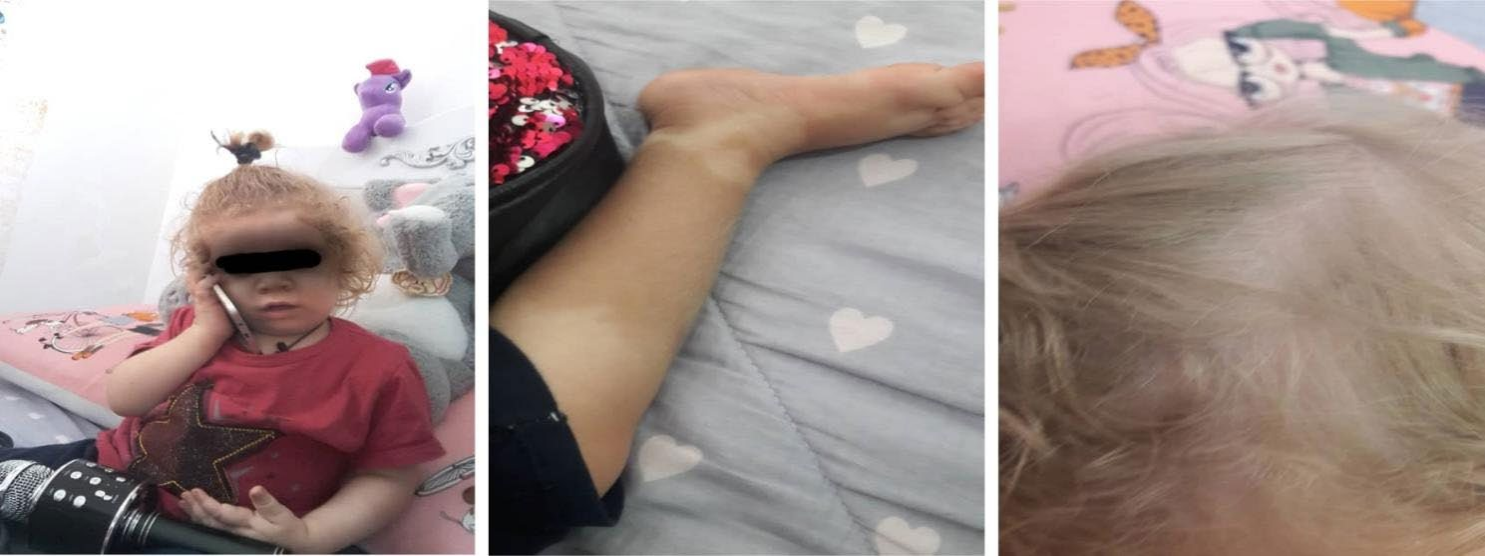
The clinical manifestation of the patient

At the age of 27 months, the patient was referred to the rheumatology clinic due to several episodes of high grade fever, inflammatory pericardial effusion, and active left knee arthritis with an increase in inflammatory marker levels without any localized origin, as well as an erythematous plaque on the leg and a history of multiple hospitalizations. A skin biopsy revealed septal panniculitis compatible with erythema nodosum. The patient was thoroughly evaluated in terms of rheumatology, her height, and weight were 82 cm and 10 kg in physical examination, respectively. We found blonde hair, frontal bossing, and macular rashes on limbs, and eventually, according to the lack of response to treatment and the history of respiratory infections, an immunological examination including CBC, CD markers, and immunoglobulins levels was performed. The clinical manifestations of the patient are shown in Fig. 1.

Low serum IgG levels, decreased CD4/CD8 ratio and CD27 level, and a poor response to candida in the lymphocyte transformation test (LTT) were detected (Table 1). Intravenous immunoglobulin (IVIG) and prophylactic antibiotics were started which led to the control of the patient’s fever and other symptoms.

**Table 1:** The results of hematological, biochemical and immunological tests of the patient

Test	Value	Normal Range	Test	Value	Normal Range
**WBC(cell/mm** ^3^ **)**	8900	6000–17,000	**IgM ( mg/dl)**	59	40–150
**Neutrophil(cell/mm** ^3^ **)**	4450	1500–8500	**IgG( mg/dl)**	520	600–1100
**Lymphocyte(cell/mm** ^3^ **)**	3604	3000–9500	**IgA ( mg/dl)**	21	51–297
**Hb(g/dl)**	7.4	11.5–13.5	**IgE ( IU/ml)**	1	Up to 144
**PLT**	566,000	150,000–450,000	**CD3%(total count)**	65.2 (3338)	35–78%
**ANA**	Negative	NL < 1/160	**CD4%(total count)**	33 (1690)	22–62%
**RF**	Negative	Neg < 10	**CD8%(total count)**	32.3 (1654)	12–36%
**Anti Ds DNA**	Negative	Neg < 2.6	**CD19%(total count)**	11 (563)	3–14%
**HLAB51**	Negative		**CD20%(total count)**	13.2 (676)	3–15%
**Anti CCP**	Negative	Neg < 0.3	**CD16%(total count)**	15.4 (229)	3–5%
**Anti Phospholipid Ab**	Negative	Negative	**CD56%(total count)**	16.2	3–5%
**Anti SSA**(RO)	Negative	Neg < 15	**CD4/CD8**	1.02	1.5-2
**Anti SSB**(La)	Negative	Neg < 20	**CD27%**	3.90	9–35%
**ESR (mm/hr)**	57 (H)	Nl < 20	**LTT(PHA)**	7.1	Nl˃3.5
**CRP (mg/L)**	43	Nl < 6	**LTT (Candida)**	1.5	Nl˃2.5
**Anti-Tetanus Ab**(IU/ml)	1.57	˃0.1	**LTT (BCG**)	6.2	Nl˃2.5
**Anti-Diphtheria Ab**(IU/ml)	0.29	˃0.01			

WBC: White Blood cell, Hb: Hemoglobin, PLT: Platelet, ANA: AntiNuclear Antibody, RF: Rheumatoid Factor, Anti Ds DNA: Anti Double stranded DNA, Anti CCP: Anti Cyclic Citrullinated Peptide, Anti SSA Ab: anti–Sjögren’s-syndrome-related antigen A autoantibody, Anti SSB: anti–Sjögren’s-syndrome-related antigen B autoantibody, ESR: Erythrocyte Sedimentation Rate, CRP: C-reactive Protein, Ig: Immunoglobulin, LTT: lymphocyte transformation test, PHA: Phytohemagglutinin, BCG: Bacillus Calmette-Guerin

After about 5 months of the follow-up, the patient was hospitalized due to polyuria, polydipsia, tachypnea, and lethargy with the diagnosis of diabetic ketoacidosis and was discharged with injectable insulin. Whole-exome sequencing was performed on the patient’s whole blood sample. Variant interpretation of interested variants was accomplished through the American College of Medical Genetics and Genomics (ACMG). A novel heterozygous variant (c.1429 G > A; p.Glu477Lys) was found in the PIK3CD gene (Table 2). The variant was validated in the patient, and segregation analysis showed the mother is the carrier for the variant. According to the ACMG guideline, this variant can be classified as a Variant of Unknown Significance (VUS).

**Table 2:** The result of genetic sequencing of the patient

Gene/Transcript	Variant Location	Chromosome position (GRCh37)	Relationship with the patient	Zygosity	Variant classification
PIK3CD ENST00000377346.4 NM-005026	Exon11	c.1429G > A p.E477K	Proband	Het	VUS
			Mother	Het	
			Father	N	

## Discussion

In this report, we described a girl with a novel mutation in the PIK3CD gene. She had a recurrent fever, and erythema nodosum with the manifestations of combined immunodeficiency in immunological investigations. She experienced several episodes of viral and bacterial infections, autoimmune disorders (hypothyroidism and type 1 diabetes mellitus), and auto-inflammatory manifestations (left knee arthritis, pericardial effusion).

Frequent infections were considered the main manifestation of primary immune deficiencies, but in recent years, non-infectious manifestations related to this group of diseases have received more attention. These can be the first complaint for a patient to visit the doctor [9].

In the largest cohort study on 53 patients with this type of immunodeficiency conducted by Tanya I. Coulter et al., upper and lower respiratory tract infections were the most common symptoms, which were seen in 98% of patients. Our patient has suffered from recurrent bacterial sinusitis and pneumonia since early infancy. The autoimmune or inflammatory diseases were reported in 34% of the patients. Among them, only 3 patients had autoimmune thyroid disease in adulthood, two patients suffered from arthritis, and one patient had pericardial effusion, but our patient experienced all three of these very rare manifestations in infancy. Global developmental or isolated speech delays were detected in 19% of patients [10]. According to the results of this study, our patient also had motor and verbal development delays.

Frequent respiratory infections can lead to lung damage and bronchiectasis, so it is very important to pay attention to this point in the follow-up of patients. Quick and timely diagnosis can prevent such serious injuries in patients [11]. Nonmalignant lymphoproliferative manifestations are among the common manifestations in patients, but they were not seen in our patient [12].

Considering the susceptibility of these patients to chronic viral infections such as Epstein Barr Virus (EBV), the risk of cancer in them is higher than in the normal population [13]. Fortunately, our patient has not been infected with chronic viral infections such as Epstein’s, etc., but we should always keep this point in mind during follow-up.

Immunodeficiency may be accompanied by cardiac tamponade; however, it is not common. Farouji et al. reported a 35-year-old woman with immune deficiency presented with cardiac tamponade [14]. Our patient had a massive pericardial effusion that could have led to tamponade, but her early diagnosis saved her life.

Early diagnosis and management of a patient with PI3KCD deficiency are important.

Somatic and germline mutations in genes involved in the PI3Kd pathway can result in different clinical and laboratory manifestations. Somatic mutations are associated with cancer. Both overactivation and underactivation of PI3Kd as a result of germline mutations could lead to immunodeficiency and immune dysregulation. Recently, more than 200 such patients have been detected [13]. The immunologic workups of our patient were compatible with combined immunodeficiency. Besides, she had manifestations of immune dysregulation.

To our knowledge, the variant of the PIK3CD gene (c.1429 G > A) found in this study has not previously been reported. Lu et al. reported two cases with frequent pulmonary infections, sinusitis, and a positive anti-neutrophil cytoplasmic antibody (ANCA). The whole exome sequencing revealed a Glu1021Lys (c.3061 G > A) mutation in the PIK3CD gene [15]. Moreover, a fourteen-year-old girl reported by Nichols-Vinueza et al. had a PIK3CD mutation (c.1546 G > A) and a history of severe atopy, saddle nose deformity, nasal septal perforation, repeated infections, and increased IgE and eosinophilia [16]. Further, she had a mid-face deformity and recurrent infections compared with our patient. It seems that mutations in PIK3CD are accompanied by midface malformations. Moreover, our report showed that a novel variant in *PIK3CD* gene in exon 11 related to recurrent infection as well as some facial and dermal involvement. Antibiotic prophylaxis and immunoglobulin replacement therapy (intravenous immunoglobulin or subcutaneous immunoglobulin) are among the main treatments in most patients. Bacterial infections were decreased in 89% of patients in the Elkaim et al. APDS2 cohort with long-term IRT, and 61% of patients received antibiotic prophylaxis [17].

Sirolimus, the mTOR inhibitor, appears to be effective in controlling lymphoproliferative symptoms in these patients [18]. The findings suggests that HSCT is curative in younger patients with life threatening complications of APDS [19].

PI3Kδ inhibitors are new drugs recently used in the treatment of this group of patients. They have had satisfactory results in resolving lymphoproliferation, but it seems that they do not replace a hematopoietic stem cell transplant [20, 21].

In our practice, we performed supportive treatments for the patient, like IVIG infusions and antibiotic therapy. Considering the poor outcome of this disease, bone marrow transplantation is one of the decisions for the patient; however, she has not had a proper, fully HLA matched donor yet.

## Conclusion

The variant in *PIK3CD* (c.1429 G > A) is a novel mutation that can cause immunodeficiency in patients. This gene can be presented at an early age. Whole-exome sequencing is a valuable method for diagnosing patients with an immunodeficiency that physicians do not have any direct clue about. The exact genetic diagnosis would be helpful for controlling the disease and providing appropriate treatment.

## Data Availability

Not applicable.

## Abbreviations

PI3K: Phosphoinositide 3-kinase
APDS: Activated phosphoinositide 3-kinase delta syndrome
*PIK3CD*: *Phosphatidylinositol-4, 5-bisphosphate 3-kinase, catalytic subunit delta*
PID: Primary immune deficiency
FTT: Failure to thrive
ICU: Intensive care unit
LTT: Lymphocyte transformation test
IVIG: Intravenous immunoglobulin
ACMG: American College of Medical Genetics and Genomics
VUS: Variant of Unknown Significance
ANCA: Anti-neutrophil cytoplasmic antibody
WBC: White Blood cell
Hb: Hemoglobin
PLT: Platelet
ANA: AntiNuclear Antibody
RF: Rheumatoid Factor
Anti Ds DNA: Anti Double stranded DNA
Anti CCP: Anti Cyclic Citrullinated Peptide
Anti SSA Ab: anti–Sjögren’s-syndrome-related antigen A autoantibodies
Anti SSB: anti–Sjögren’s-syndrome-related antigen B autoantibodies
ESR: Erythrocyte Sedimentation Rate
CRP: C-reactive Protein
Ig: Immunoglobulin
PHA: Phytohemagglutinin
BCG: Bacillus Calmette-Guerin
